# Comparison of diagnosis-based risk adjustment methods for episode-based costs to apply in efficiency measurement

**DOI:** 10.1186/s12913-023-10282-4

**Published:** 2023-12-01

**Authors:** Juyoung Kim, Minsu Ock, In-Hwan Oh, Min-Woo Jo, Yoon Kim, Moo-Song Lee, Sang-il Lee

**Affiliations:** 1 Department of Preventive Medicine, University of Ulsan College of Medicine, 88 Olympic-Ro 43-Gil, Songpa-Gu, Seoul, 05505 Republic of Korea; 2 Department of Preventive Medicine, School of Medicine, Kyung Hee University, Seoul, Republic of Korea; 3 Department of Health Policy and Management, Seoul National University College of Medicine, Seoul, South Korea

**Keywords:** Charlson Comorbidity Index (CCI), Episode-based costs, Hierarchical Condition Categories (HCCs), Korean Diagnostic Related Group (KDRG), Risk adjustments

## Abstract

**Background:**

The recent rising health spending intrigued efficiency and cost-based performance measures. However, mortality risk adjustment methods are still under consideration in cost estimation, though methods specific to cost estimate have been developed. Therefore, we aimed to compare the performance of diagnosis-based risk adjustment methods based on the episode-based cost to utilize in efficiency measurement.

**Methods:**

We used the Health Insurance Review and Assessment Service–National Patient Sample as the data source. A separate linear regression model was constructed within each Major Diagnostic Category (MDC). Individual models included explanatory (demographics, insurance type, institutional type, Adjacent Diagnosis Related Group [ADRG], diagnosis-based risk adjustment methods) and response variables (episode-based costs). The following risk adjustment methods were used: Refined Diagnosis Related Group (RDRG), Charlson Comorbidity Index (CCI), National Health Insurance Service Hierarchical Condition Categories (NHIS-HCC), and Department of Health and Human Service-HCC (HHS-HCC). The model accuracy was compared using R-squared (R^2^), mean absolute error, and predictive ratio. For external validity, we used the 2017 dataset.

**Results:**

The model including RDRG improved the mean adjusted R^2^ from 40.8% to 45.8% compared to the adjacent DRG. RDRG was inferior to both HCCs (RDRG adjusted R^2^ 45.8%, NHIS-HCC adjusted R^2^ 46.3%, HHS-HCC adjusted R^2^ 45.9%) but superior to CCI (adjusted R^2^ 42.7%). Model performance varied depending on the MDC groups. While both HCCs had the highest explanatory power in 12 MDCs, including MDC P (Newborns), RDRG showed the highest adjusted R^2^ in 6 MDCs, such as MDC O (pregnancy, childbirth, and puerperium). The overall mean absolute errors were the lowest in the model with RDRG ($1,099). The predictive ratios showed similar patterns among the models regardless of the  subgroups according to age, sex, insurance type, institutional type, and the upper and lower 10th percentiles of actual costs. External validity also showed a similar pattern in the model performance.

**Conclusions:**

Our research showed that either NHIS-HCC or HHS-HCC can be useful in adjusting comorbidities for episode-based costs in the process of efficiency measurement.

**Supplementary Information:**

The online version contains supplementary material available at 10.1186/s12913-023-10282-4.

## Background

Health spending as a share of gross domestic product (GDP) has gradually increased during the last 15 years, from 7.8% in 2005 to 8.8% in 2020 among the Organisation for Economic Cooperation and Development (OECD) countries [1]. The estimates reported 10.2% of GDP in 2030, a far higher value compared with the current proportion [2]. The rise in healthcare expenditures impacts the affordability of individual patients and payers. The shares of GDP spent on health positively correlate with catastrophic payments connected to affordability [3]. In addition, a continuous increase in health spending can inhibit the achievement of universal health coverage, which is a target under the United Nations Sustainable Development Goal 3 (Ensure healthy lives and promote well-being for all at all ages) [4]. In South Korea, the annual health expenditure covered by the National Health Insurance Service has been on the rise in the last two decades ($80 hundred million in 2000 to $65 billion in 2021) [5]. Worldwide health expenditure is expected to accelerate due to aging societies and technological advancement. In particular, due to an oversupply of services, sustainable health financing can deteriorate more in countries adopting a fee-for-service payment system, such as South Korea [6].

The rising health spending led to increased interest in efficiency in the quality of care. The efficiency measurement has progressed from measuring the amount of service provided (e.g., length of stay or physician visits) to calculating the ratio between observed and predicted costs [7]. The use of predicted costs in the efficiency measurement only allows comparability by adjusting for risk factors contributing to differences in the outcome of interest, such as sociodemographic factors or comorbidities. A comorbidity risk adjustment method for mortality, such as Charlson Comorbidity Index (CCI), has been widely used in clinical but also health expenditure research [8–10]. However, the choice of risk adjustment method should be based on the outcome of interest, which is closely related to the selection of the model’s construction and statistical techniques [11]. The United States Center for Medicare and Medicaid Services (CMS) introduced Hierarchical Condition Categories (HCC, CMS-HCC) for cost estimation. The CMS-HCC has been recently utilized in value-based payments such as the Merit-based Incentive Payment Systems or Hospital Value-Based Purchasing [12, 13]. In addition, the US health insurance system has started using another version of HCC, the Department of Health and Human Service-HCC (HHS-HCC), which is related to risk selection on the premiums under the Affordable Care Act [14].

In South Korea, there have been efforts to utilize a risk adjustment method specific to costs by adopting the National Health Insurance Service-HCC (NHIS-HCC), which is a modified version of CMS-HCC based on the annual cost estimation [15, 16]. In a recent study, the NHIS-HCC was utilized to estimate episode-based costs in the process of efficiency measurement [17]. However, studies have yet to evaluate the feasibility of the NHIS-HCC based on episode-based costs by comparing it with currently available risk adjustment methods. In addition, the disease groups in the NHIS-HCC are limited to the elderly because the CMS-HCC was developed for use in Medicare that targets people 65 or older [16, 18]. On the other hand, the HHS-HCC includes more various disease groups, including pregnancy, delivery, and neonate-related diseases [19].

Therefore, this study aimed to compare the diagnosis-based risk adjustment methods, including the mortality adjustment tool (i.e., CCI), risk-adjusted Diagnosis-Related Group (DRG), and HCCs, based on episode-based costs in the context of efficiency measurement.

## Methods

### Data sources

We used the Health Insurance Review and Assessment Service-National Patient Sample (HIRA-NPS), which is the representative claims database that randomly samples 3% of the annual beneficiaries in South Korea [20]. We used the 2018 HIRA-NPS for model evaluation, which was the latest available dataset at the design of the study. For external validity, we used the 2017 dataset considering the cross-sectional feature of HIRA-NPS and the sample size for regression [21].

### Episode construction specifications

**Fig. 1:**
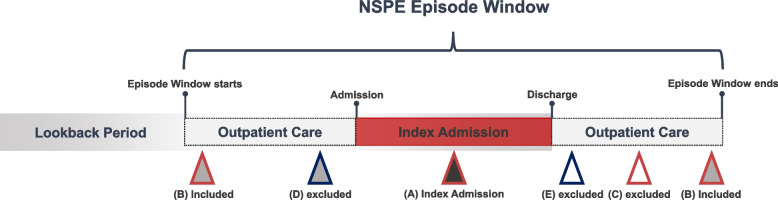
NSPE episode framework. (**A**) Index admission, (**B**) Identical primary diagnostic code (3 digits) and institution compared to the index admission, (**C**) Non-identical primary diagnostic code (3 digits) but the same institution compared to the index admission, (**D**) Identical primary diagnostic code (3 digits) but non-identical institution compared to the index admission, (**E**) Non-identical primary diagnostic code (3 digits) and institution compared to the index admission. NSPE, National Health Insurance Service Spending Per Episode

We adopted the episode definition used in the National Health Insurance Service Spending Per Episode (NSPE) index, an episode-based efficiency measure for hospitals (Fig. 1) [17]. An NSPE episode includes actual hospitalization (i.e., index admission) and the related outpatient services during the episode window (before and after the admission), reflecting the shifting services from inpatient to outpatient settings [22]. First, we create index admission datasets using annual claims data (i.e., 2017 and 2018 HIRA-NPS) from April to November, considering the definition of the NSPE episode and the lookback period to obtain comorbidity information. Exclusion criteria for index admission were as follows: (1) length of stay ≤ 1 day, (2) cost for index admission ≤ $0, and (3) error DRGs.

The NSPE window starts 30 days before the admission date and ends after 30 days following the discharge date. We only assigned related outpatient services to the NSPE episodes during the episode window. Related outpatient services are defined as the same primary diagnostic code (3 digits) and the same institution as the index admission. Considering the overlap between episode windows, we adjusted overlapped episodes depending on the types of overlapping: (1) a single episode (no adjustment), (2) multiple episodes, no overlap (no adjustment), (3) multiple episodes, overlapping but distinct periods (no adjustment), (4) multiple episodes, overlapping and non-distinct periods (adjusted by assigning half of the overlapped periods to pre- and post-episodes, respectively) (Additional file 1) [17]. A lookback period for comorbidities included episode windows and the previous two months from the episode window.

### Model estimation and performance evaluation

We estimated the current episode costs (i.e., concurrent model) using a linear regression by the Major Diagnostic Categories (MDCs) [23, 24]. We used the ordinary least squares (OLS) regression, practically used to estimate episode-based costs [25–27]. Considering the requirement of 10 observations for each additional explanatory variable for the regression, as a rule of thumb, we screened the number of episodes according to the MDC groups [28]. As for MDCs not satisfying the minimum number of observations for the regression, several MDC groups were merged based on similarities; otherwise, we inevitably excluded those MDCs from the analysis due to a lack of observation for the estimation. We merged MDCs as follows: MDC ST (Infectious and Parasitic Diseases), MDC S (Infectious and Parasitic Diseases: HIV) and MDC T (Infectious and Parasitic Diseases); MDC UV (Mental Diseases and Disorders), MDC U (Mental Diseases and Disorders) and MDC V (Alcohol/Drug Use and Alcohol/Drug Induced Organic Mental Disorders); MDC WXY (Trauma, Injuries, Poisoning and Burns), MDC W (Multiple Trauma), MDC X (Injuries, Poisoning and Toxic Effects of Drugs), MDC Y (Burns) (Additional file 2). We excluded MDC A (PreMDC, transplants and tracheostomy DRGs), MDC Q (Disease and Disorders of the Blood-Forming Organs and Immunological Disorders), and MDC Z (Factors Influencing Health Status and Other Contacts with Health Services) from the analysis due to the insufficient number of observations within the MDC.

The dependent variable in the regression analysis was the total expenditure for inpatient and outpatient services during the individual NSPE episode window, obtained by the National Health Insurance Service (NHIS). Considering skewed distribution, we used winsorized NSPE episode costs as the dependent variable for the regression analysis. We obtained NSPE episode costs from the claims by the NHIS, a single insurer providing health insurance in South Korea. Therefore, the NSPE episode costs included the amount paid by the NHIS and a portion of the out-of-pocket costs (only statutory payment but not non-payment items). 

Winsorizing was adopted to treat outliers at the 0.5 percentile (upper and lower bounds), considering the average cost per day ($180) in 2018 from claims statistics and the average NSPE episode cost by MDCs ($30–$202) [29] (Additional file 3). We used costs in South Korean Won (KRW) in the model estimation, then converted and presented them to United States Dollars (USD) using annual average exchange rates at the time of the datasets (2017, 1 USD = 1,130,48 KRW; 2018, 1 USD = 1,100.58 KRW) [30].

The explanatory variables included age groups (age 0–2, age 3–19, age 20–39, age 40–59, age 60 and over), sex, insurance type (National Health Insurance, Medical Aid), type of institution (tertiary hospital, general hospital, and hospital), Adjacent Diagnosis Related Group (ADRG), and diagnosis-based risk adjustment. Due to the limitation of the categorical age variable in the HIRA-NPS and the observation for explanatory variables, we collapsed age groups as follows: (1) age 0–2, infants and toddlers, (2) age 3–19, child and teenage, (3) age 20–39, young adults, (4) 40–59, middle-aged adults, (5) age 60 and over, older adults. Depending on the risk adjustment for comorbidities, we constructed five separate models: (1) No risk adjustment (Model 0), (2) Refined Diagnosis Related Group (RDRG, Model 1) [23], (3) CCI (Model 2) [31, 32], (4) NHIS-HCC (Model 3) [15–17], (5) HHS-HCC (Model 4) [14, 33].

The model performance at the episode level was evaluated using R-squared (R^2^) and adjusted R^2^ (adj. R^2^) statistics according to the MDC groups [34]. We also measured the Mean Absolute Errors (MAEs) to compare the average magnitude of the errors between observed and predicted values [24]. The predictive ratio (PR) was used to compare the accuracy within subgroups (age group, sex, types of institutions, insurance types, and the highest and lowest decile of the observed costs) [14, 24]. We verified our performance comparison using HIRA-NPS 2017, the dataset separately sampled compared to the dataset used for estimation (HIRA-NPS 2018). The HIRA-NPS are cross-section data selecting different patients every year in the pursuit of privacy protection [21]. Considering the insufficient sample size to split for external validation from the annual dataset, we used another year's dataset differently selected representatively from the whole claims data.

Additionally, we conducted several sensitivity analyses to explore models dealing with the right-skewed distribution of residuals and the potential clustering effect of medical institutions. First, we used log-transformed costs in the model using HHS-HCC for comorbidity (Model 5) [11, 35]. Second, we trimmed individual datasets by MDCs using the interquartile range (IQR) to deal with outliers (Model 6) [36]. Then, we compared these two additional models with Model 4 using winsorized NSPE episode costs. Third, we examined the clustering effect using the Intracluster Correlation Coefficient (ICC) based on the Model 4 [37, 38]. Then, we conducted a multilevel analysis considering nested within institutional types (Model 7) and presented model fits (Akaike Information Criterion, AIC; Schwarz’s Bayesian Information Criterion, BIC; Pseudo-R^2^) [39].

### Efficiency measurement

Considering the purpose of cost estimation for efficiency measurement in this study, we compared the descriptive statistics and the distribution of the NSPE indexes, a modified version of the Medicare Spending Per Beneficiary measure [13], using estimates from individual models. The steps to calculate the NSPE indexes were as follows: (1) calculating observed and predicted costs of individual NSPE episodes, (2) treatment of outliers, (3) calculating average observed and predicted NSPE costs of the individual institution, (4) calculating the NSPE ratio as observed mean to predicted mean of costs, (5) calculating NSPE amount by multiplying the average observed costs and NSPE ratios, (6) deriving NSPE indexes of individual institutions as a ratio with weighted median NSPE amounts [17].

This research using administrative data was deemed exempt from review by the Asan Medical Center Institutional Review Board (#2021–0093). All analyses were conducted using SAS 9.4 (SAS Institute, Cary, NC, USA).

## Results

### Episode description

The original dataset consisted of 147,493 episodes for the estimation (HIRA-NPS 2018) and 144,877 for the external validation (HIRA-NPS 2017) (Table 1). After excluding the MDCs not satisfying an appropriate number of observations for regression analysis, episode counts were 145,792 and 143,158 in 2018 and 2017, respectively. The 2018 dataset included 106,876 beneficiaries and 1,772 institutions. The mean (standard deviation, SD) inpatient days was 8.2 (10.0). In the 2017 dataset, the number of beneficiaries and institutions was 104,736 and 1,763, respectively; the mean (SD) of inpatient days was 8.3 (10.2).

**Table 1:** Episode distribution according to MDC

MDC	2017		2018	
	**Original data,** **N (%)**	**Selected data,** **N (%)**	**Original data,** **N (%)**	**Selected data,** **N (%)**
A	170 (0.1)	0 (0.0)	160 (0.1)	0 (0.0)
B	10,972 (7.6)	10,972 (7.7)	11,038 (7.5)	11,038 (7.6)
C	1,664 (1.1)	1,664 (1.2)	1,705 (1.2)	1,705 (1.2)
D	9,107 (6.3)	9,107 (6.4)	9,134 (6.2)	9,134 (6.3)
E	15,372 (10.6)	15,372 (10.7)	15,750 (10.7)	15,750 (10.8)
F	6,536 (4.5)	6,536 (4.6)	6,700 (4.5)	6,700 (4.6)
G	21,528 (14.9)	21,528 (15.0)	21,621 (14.7)	21,621 (14.8)
H	7,571 (5.2)	7,571 (5.0)	7,636 (5.2)	7,636 (5.2)
I	33,107 (22.9)	33,107 (23.1)	34,422 (23.3)	34,422 (23.6)
J	5,357 (3.7)	5,357 (3.7)	5,075 (3.4)	5,075 (3.5)
K	3,072 (2.1)	3,072 (2.1)	3,213 (2.2)	3,213 (2.2)
L	6,319 (4.4)	6,319 (4.4)	6,495 (4.4)	6,495 (4.5)
M	1,076 (0.7)	1,076 (0.8)	1,153 (0.8)	1,153 (0.8)
N	4,013 (2.8)	4,013 (2.8)	3,840 (2.6)	3,840 (2.6)
O	5,334 (3.7)	5,334 (3.7)	5,081 (3.4)	5,081 (3.5)
P	1,775 (1.2)	1,775 (1.2)	2,082 (1.4)	2,082 (1.4)
Q	802 (0.6)	0 (0.0)	751 (0.5)	0 (0.0)
R	3,554 (2.5)	3,554 (2.5)	3,883 (2.6)	3,883 (2.7)
S	16 (0.0)	2,425 (1.7)	25 (0.0)	2,555 (1.8)
T	2,409 (1.7)		2,530 (1.7)	
U	1,307 (0.9)	1,682 (1.2)	1,362 (0.9)	1,657 (1.1)
V	375 (0.3)		295 (0.2)	
W	681 (0.5)	2,694 (1.9)	650 (0.4)	2,752 (1.9)
X	1,652 (1.1)		1,783 (1.2)	
Y	361 (0.2)		319 (0.2)	
Z	747 (0.5)	0 (0.0)	790 (0.5)	0 (0.0)
Total	144,877 (100.0)	143,158 (100.0)	147,493 (100.0)	145,792 (100.0)

*MDC* Major diagnostic category

NSPE episodes' characteristics in each MDC are presented in Table 2. MDC UV had the longest mean length of stay (20.6 days), whereas MDC C had the shortest mean length (3.9 days). Overall, Emergency Room (ER) episodes consisted of 19.7%: the proportion of ER episodes was the highest in MDC WXY (42.6%) and the lowest in MDC P (6.2%). The total numbers of ADRG and RDRG types were 1,164 and 2,933, respectively. While MDC I had the most types of ADRGs (*n* = 145) and RDRGs (*n* = 387), MDC UV and MDC P had the fewest types of ADRGs (*n* = 15) and RDRGs (*n* = 26), respectively. In particular, the number of ADRGs and RDRGs was the same in MDC P, implying no risk adjustment of comorbidities. The average cost of the NSPE episode was $2,422, with an average of $2,308 for inpatient care and $115 for outpatient care. While MDC F showed the highest mean costs in inpatient ($4,807) and NSPE episodes ($4,857), outpatient costs were the highest in MDC J ($374). On the other hand, MDC D had the lowest mean costs in inpatient ($1,019) and NSPE episodes ($1,104); outpatient costs were the lowest in MDC P ($9). The average number of diagnostic codes for comorbidities per episode was 16.9. The mean number of codes for comorbidities was the largest in MDC P (48.4) and the smallest in MDC O (8.2).

**Table 2:** General characteristics of NSPE episodes

MDC	Episode counts^a^	Inpatient days^b^	Admission via ER^a^	ADRG^c^	RDRG^c^	Inpatient costs^bd^	Outpatient costs^bd^	NSPE episode costs^bd^	Number of comorbidities^b^
B	11,038 (7.6)	11.2 (17.6)	3,265 (29.6)	157	415	3,266 (5,457)	52 (373)	3,319 (5,489)	17.7 (11.7)
C	1,705 (1.2)	3.9 (3.0)	179 (10.5)	48	88	1,850 (1,132)	133 (356)	1,983 (1,208)	16.4 (10.2)
D	9,134 (6.3)	4.9 (4.0)	1,598 (17.5)	90	185	1,019 (1,544)	85 (383)	1,104 (1,671)	13.8 (9.0)
E	15,750 (10.8)	8.7 (9.1)	3,445 (21.9)	71	257	2,182 (3,755)	107 (628)	2,289 (3,855)	17.8 (11.5)
F	6,700 (4.6)	7.0 (9.2)	2,317 (34.6)	109	219	4,807 (7,404)	50 (100)	4,857 (7,410)	20.0 (13.0)
G	21,621 (14.8)	6.0 (6.6)	5,340 (24.7)	106	285	1,872 (2,707)	101 (396)	1,974 (2,810)	15.5 (11.5)
H	7,636 (5.2)	9.0 (9.1)	2,355 (30.8)	65	158	3,571 (5,344)	188 (566)	3,759 (5,415)	19.3 (12.0)
I	34,422 (23.6)	9.7 (9.4)	2,989 (8.7)	145	387	1,952 (2,505)	81 (190)	2,033 (2,531)	16.2 (11.2)
J	5,075 (3.5)	7.0 (8.5)	595 (11.7)	40	97	1,744 (2,481)	374 (988)	2,118 (2,896)	15.2 (11.0)
K	3,213 (2.2)	8.2 (8.8)	601 (18.7)	46	115	2,049 (2,504)	127 (285)	2,175 (2,551)	19.2 (13.0)
L	6,495 (4.5)	8.3 (8.6)	2,049 (31.5)	71	234	2,521 (3,479)	254 (720)	2,775 (3,579)	20.1 (12.6)
M	1,153 (0.8)	6.6 (7.7)	116 (10.1)	29	56	1,946 (2,595)	244 (539)	2,190 (2,672)	16.9 (10.7)
N	3,840 (2.6)	5.4 (4.8)	365 (9.5)	45	96	2,293 (2,090)	248 (679)	2,541 (2,232)	13.2 (9.0)
O	5,081 (3.5)	5.3 (4.8)	575 (11.3)	33	71	1,628 (1,108)	20 (68)	1,647 (1,113)	8.2 (5.0)
P	2,082 (1.4)	7.3 (9.9)	130 (6.2)	26	26	2,632 (8,540)	9 (53)	2,641 (8,565)	48.4 (37.2)
R	3,883 (2.7)	7.3 (9.9)	389 (10.0)	18	54	3,521 (7,264)	286 (946)	3,807 (7,471)	19.4 (11.0)
ST	2,555 (1.8)	7.6 (10.7)	926 (36.2)	22	62	2,019 (4,997)	16 (61)	2,035 (4,997)	15.9 (12.7)
UV	1,657 (1.1)	20.6 (26.7)	286 (17.3)	15	52	2,592 (3,944)	74 (169)	2,666 (3,970)	14.9 (12.7)
WXY	2,752 (1.9)	10.6 (11.7)	1,172 (42.6)	28	76	2,410 (3,723)	59 (127)	2,470 (3,725)	15.2 (11.9)
Total	145,792 (100.0)	8.2 (10.0)	28,692 (19.7)	1,164	2,933	2,308 (3,953)	115 (466)	2,422 (4,020)	16.9 (12.8)

^a^n (%); ^b^Mean (SD); ^c^Number of types; ^d^Unit: United States Dollar (USD), converted from South Korean Won (KRW) (1 USD = 1,100.58 KRW, 2018)

*ADRG* Adjacent Diagnosis Related Group, *ER* Emergency Room, *MDC* Major Diagnostic Category, *NSPE* National Health Insurance Service Spending Per Episode, *RDRG* Refined Diagnosis Related Group

### Model fit

The overall mean of R^2^ (41.6%) and adjusted R^2^ (adj. R^2^ 40.8%) from MDC groups were the lowest in Model 0, which was non-risk-adjusted for comorbidities (Table 3). While using risk adjustment methods for comorbidities improved the performance compared to Model 0 in all models, the amount of improvement differed depending on the risk adjustment methods used. Model 2 using CCI (adj. R^2^ 42.7%) showed a minor improvement over Model 0 (△1.9%), but it was inferior to other risk-adjusted models (Model 1, Model 3, Model 4). Although Model 1, including RDRG (adj. R^2^ 45.8%), was superior to both Model 0 and Model 2, models using HCCs showed better performance than Model 1 (Model 3 adj. R^2^ 46.3%, Model 4 adj. R^2^ 45.9%). Model 3, risk-adjusted with NHIS-HCC, had the highest explanatory power among the five models. The trends mentioned above of model performance did not significantly change in the weighted means considering episode counts, as Model 3 and Model 4 (using HCCs) showed superiority in the explanatory power (Model 3 weighted adj. R^2^ 51.0%, Model 4 weighted adj. R^2^ 50.7%).

**Table 3:** R^2^ (%) and adjusted R^2^ (%) of models

Model	R^2^							Adjusted R^2^						
	**Mean** **(SD)**	**Min**	**Q1**	**Median**	**Q3**	**Max**	**Wt mean(SD)**	**Mean** **(SD)**	**Min**	**Q1**	**Median**	**Q3**	**Max**	**Wt mean** **(SD)**
Mode 0 (ADRG)	41.6 (14.6)	9.1	34.0	41.6	49.2	77.5	46.2 (10.8)	40.8 (14.9)	7.7	32.9	41.2	48.0	77.1	45.7 (11.1)
Model 1 (RDRG)	47.3 (13.4)	13.7	40.8	46.8	53.6	77.5	52.0 (10.3)	45.8 (14.0)	10.7	39.0	46.1	51.8	77.1	50.9 (10.8)
Model 2 (ADRG + CCI)	43.7 (14.1)	10.5	36.6	44.5	50.3	77.5	47.9 (10.3)	42.7 (14.5)	8.5	35.6	43.5	49.1	77.1	47.3 (10.6)
Model 3 (ADRG + NHIS-HCC)	47.9 (13.3)	14.6	41.8	47.8	54.2	78.8	51.9 (9.9)	46.3 (14.1)	10.1	40.4	46.2	52.8	78.4	51.0 (10.5)
Model 4 (ADRG + HHS-HCC)	47.7 (13.4)	16.8	41.3	46.9	53.4	81.3	51.7 (10.2)	45.9 (14.2)	12.1	39.2	45.3	52.0	80.8	50.7 (10.8)

*ADRG* Adjacent Diagnosis Related Group, *HHS-HCC* Department of Health and Human Service Hierarchical Condition Category, *NHIS-HCC* National Health Insurance Service Hierarchical Condition Category, *RDRG* Refined Diagnosis Related Group, *R*^*2*^ R-squared, *SD* Standard Deviation, *Q1* Quartile 1, *Q3* Quartile 3, *Wt* Weighted

**Fig. 2:**
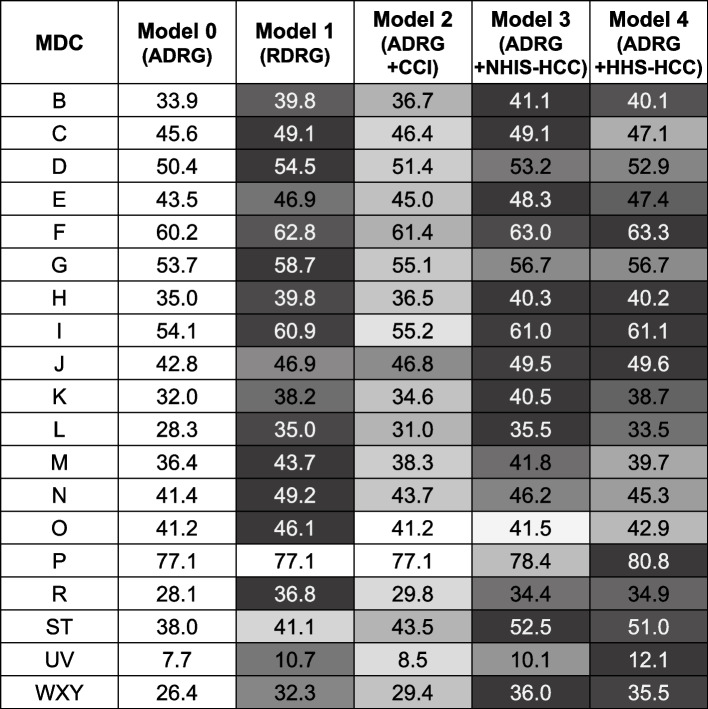
Adjusted R^2^ (%) of models according to the MDC. ADRG, Adjacent Diagnosis Related Group; CCI, Charlson Comorbidity Index; HHS-HCC, Department of Health and Human Service Hierarchical Condition Category; MDC, Major Diagnostic Category; NHIS-HCC, National Health Insurance Service Hierarchical Condition Category; RDRG, Refined Diagnosis Related Group; R^2^, R-squared

In general, model performance according to MDC groups showed similar trends among the models (Fig. 2). First, the model without risk adjustment for comorbidities had the lowest explanatory power in all MDC groups. Second, Model 2 mostly had the second lowest adj. R^2^. Third, MDC P, MDC F, and MDC I showed relatively higher performance. The explanatory powers of MDC P ranged from 77.1% to 80.8%, which are the highest among the MDC groups. MDC F (adj. R^2^ 60.2%–63.3%) and MDC I (adj. R^2^ 54.1%–61.1%) ranked second and third adj. R^2^. Lastly, the figures of explanatory power in MDC P were comparable between Model 0 (adj. R^2^ 77.1%) and Model 1 (adj. R^2^ 77.1%), implying that RDRG does not adjust for comorbidities.

**Fig. 3:**
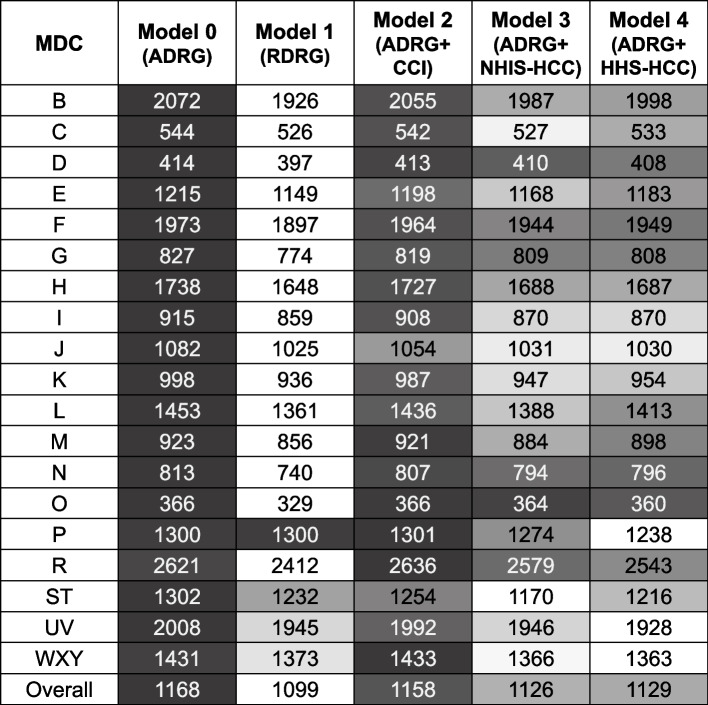
MAE of models according to the MDC. Unit: United States Dollar (USD), converted from South Korean Won (KRW) (1 USD = 1,100.58 KRW, 2018). ADRG, Adjacent Diagnosis Related Group; CCI, Charlson Comorbidity Index; HHS-HCC, Department of Health and Human Service Hierarchical Condition Category; MAE, Mean Absolute Error; MDC, Major Diagnostic Category; NHIS-HCC, National Health Insurance Service Hierarchical Condition Category; RDRG, Refined Diagnosis Related Group

Overall, MAE was superior in Model 1 using RDRG ($1,099) and inferior in Model 0 ($1,168), which was not risk-adjusted for comorbidities (Fig. 3). MAEs in individual MDC groups were also similar to the overall observation; while the values of MAE of Model 0 were the largest, they were the smallest in Model 1 in most MDCs except for MDC P, MDC ST, MDC UV, and MDC WXY. In MDC P, Model 4 using HHS-HCC ($1,238) was superior to other models; Model 0 and Model 1 had equal MAEs ($1,300), suggesting that there is no difference between the use of ADRG and RDRG. In MDC ST, Model 3 using NHIS-HCC ($1,170) had a smaller MAE than Model 1 using RDRG. In MDC UV, the MAE was the largest in Model 0 ($2,008) and the lowest in Model 4 ($1,928). While Model 4 ($1,363) presented the smallest MAE between models in MDC WXY, Model 2 ($1,433) showed the largest value. Model performance according to subgroups (sex, age group, type of medical institution, insurance type, and extreme actual costs) is shown in Table 4. In the subgroups of sex, medical institution, and insurance type, all PRs were 1.000, implying that the mean predicted costs were equal to the observed costs. In the subgroup analyses depending on the age group, the PRs were also 1.000 except for Model 1; the difference may suggest that the RDRG code embedded its unique age classification. Model 1 underestimated the group aged 60 years or older (PR 0.976) but overestimated other age groups (PR 1.011–1.105). In the actual cost groups, including both extreme values, the lower 10th percentile was overestimated (PR 3.341–3.601), and the upper 10th percentile was underestimated (PR 0.620–0.656). Additionally, estimates and values to test collinearity (Variance Inflation Factor, VIF, and Tolerance) were presented in Additional file 4.

**Table 4:** Predictive ratios of the models

Variables		Model 0 (ADRG)	Model 1 (RDRG)	Model 2 (ADRG + CCI)	Model 3 (ADRG + NHIS-HCC)	Model 4 (ADRG + HHS-HCC)
Sex	Female	1.000	1.000	1.000	1.000	1.000
	Male	1.000	1.000	1.000	1.000	1.000
Age group	0–2	1.000	1.019	1.000	1.000	1.000
	3–19	1.000	1.105	1.000	1.000	1.000
	20–39	1.000	1.039	1.000	1.000	1.000
	40–59	1.000	1.011	1.000	1.000	1.000
	60 +	1.000	0.976	1.000	1.000	1.000
Medical institution	Tertiary hospital	1.000	1.000	1.000	1.000	1.000
	General hospital	1.000	1.000	1.000	1.000	1.000
	Hospital	1.000	1.000	1.000	1.000	1.000
Insurance	NIH	1.000	1.000	1.000	1.000	1.000
	Medical aid	1.000	1.000	1.000	1.000	1.000
Actual cost	Lower 10th pct	3.601	3.357	3.472	3.354	3.341
	Middle	1.209	1.188	1.203	1.188	1.190
	Upper 10th pct	0.620	0.656	0.633	0.656	0.653

*ADRG* Adjacent Diagnosis Related Group, *CCI* Charlson Comorbidity Index, *HHS-HCC* Department of Health and Human Service Hierarchical Condition Category, *NHIS-HCC* National Health Insurance Service Hierarchical Condition Category, *NIH* National Health Insurance, *pct* percentile, *RDRG* Refined Diagnosis Related Group

**Fig. 4:**
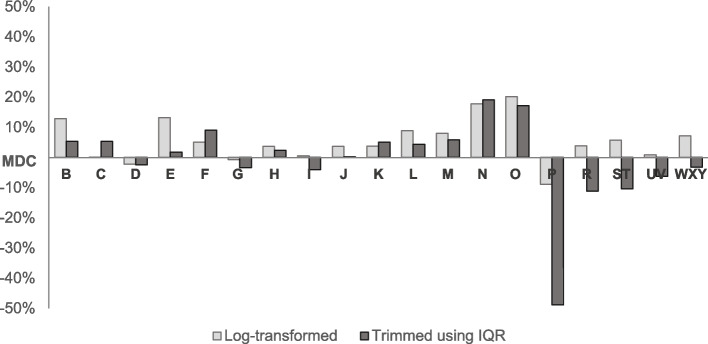
Adjusted R^2^ (%) difference depending on outlier treatment compared to winsorized costs. IQR, Interquartile Range; MDC, Major Diagnostic Category; R^2^, R-squared

In the sensitivity analyses to improve the residual distribution, the distributions were close to normal after log transformation or trimming outliers (Additional file 5). The models' explanatory power (adj. R^2^) using log-transformed cost (Model 5) or trimming costs (Model 6) improved in most MDC groups, except MDC P, MDC R, MDC ST, MDC UV, and MDC WXY (Fig. 4). In MDC P, treatment for skewed distribution dropped adj. R^2^ 8.9% (log-transformed) and 48.8% (trimmed), respectively. While log transformation improved performance (△0.8%–△7.2%), trimming decreased explanatory power (△3.2%–△11.0%) in MDC R, MDC ST, MDC UV, and MDC WXY. The results of mixed-effect models are presented in Additional file 6. The ICCs ranged between 0.018 and 0.500 in individual MDC groups. In the multilevel analysis, MDC I showed the largest AIC and BIC, whereas the lowest values were observed in MDC M.

### External validity

**Fig. 5:**
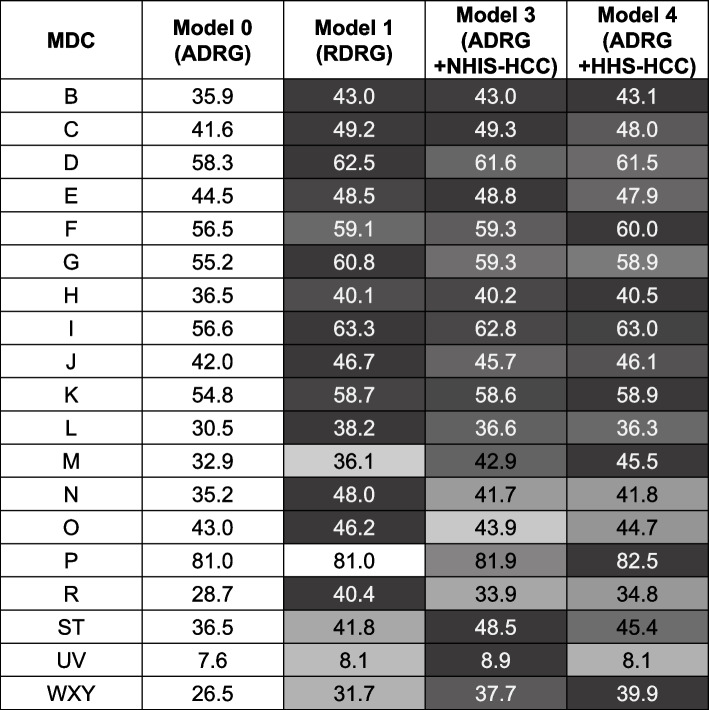
External validity, adjusted R^2^ (%) of models according to the MDC. ADRG, Adjacent Diagnosis Related Group; HHS-HCC, Department of Health and Human Service Hierarchical Condition Category; MDC, Major Diagnostic Category; NHIS-HCC, National Health Insurance Service Hierarchical Condition Category; RDRG, Refined Diagnosis Related Group; R^2^, R-squared

The overall mean value of adj. R^2^ was the lowest in Model 0 in the 2017 dataset, as in the dataset of 2018 (Model 0 adj. R^2^ 42.3%, Model 1 adj. R^2^ 47.5%, Model 3 adj. R^2^ 47.6%, Model 4 adj. R^2^ 47.7%). Model 3 using NHIS-HCC showed the highest R^2^ in the 2018 dataset, whereas the explanatory power was superior in Model 4 using HHS-HCC in the 2017 dataset. The weighted mean of adj. R^2^ also had the similar tendency (Model 0 adj. R^2^ 47.5%, Model 1 adj. R^2^ 53.1%, Model 3 adj. R^2^ 52.5%, Model 4 adj. R^2^ 52.5%). In each MDC group, the adj. R^2^ of Model 0 was inferior to those of other models (Fig. 5). The explanatory powers of MDC P (adj. R^2^ 81.0%–82.5%), MDC I (adj. R^2^ 56.6%–63.3%), and MDC F (adj. R^2^ 56.5%–60.0%) ranked the highest among the MDCs. The explanatory powers in MDC P also had the same tendency as observed in the 2018 dataset, as there was no difference in the value of explanatory power between Model 0 (adj. R^2^ 81.0%) and Model 1 (adj. R^2^ 81.0%). MDC UV had the lowest explanatory power, as seen in the 2018 dataset (adj. R^2^ 7.6%–8.9%).

**Fig. 6:**
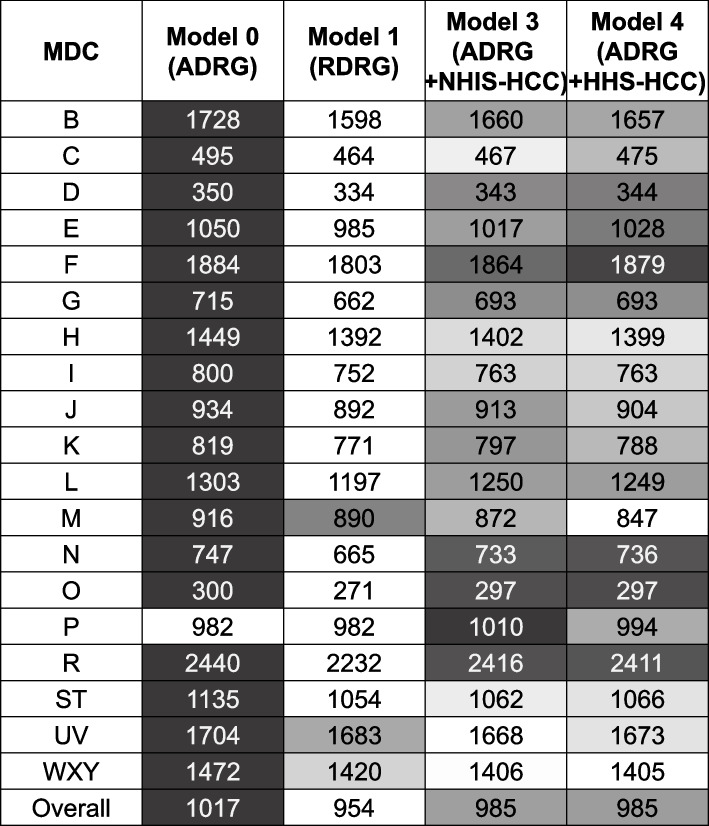
External validity, MAE of models according to the MDC. Unit: United States Dollar (USD), converted from South Korean Won (KRW) (1 USD = 1130.48 KRW, 2017). ADRG, Adjacent Diagnosis Related Group; HHS-HCC, Department of Health and Human Service Hierarchical Condition Category; MAE, Mean Absolute Error; MDC, Major Diagnostic Category; NHIS-HCC, National Health Insurance Service Hierarchical Condition Category; RDRG, Refined Diagnosis Related Group

In the validity results, overall MAEs ($954–$1,017) slightly decreased compared with the 2018 dataset ($1,099–$1,168) (Fig. 6). Model 1 showed superiority to other models in overall MAEs ($954) and MDC-specific MAEs ($271–$2,232). In MDC M, Model 4 using HHS-HCC had the smallest amount of MAE ($847) compared with other models ($872–$916). In MDC P, although Model 0 using ADRG and Model 1 using RDRG showed the lowest MAEs, RDRG did not seem to have been adjusted for comorbidities, considering the same values of adj. R^2^ between the two models. In MDC UV, Model 0 had the highest MAE ($1,704), whereas the values were lowest in Model 3 ($1,668) and Model 4 ($1,673).

### Simulation of efficiency measures

**Fig. 7:**
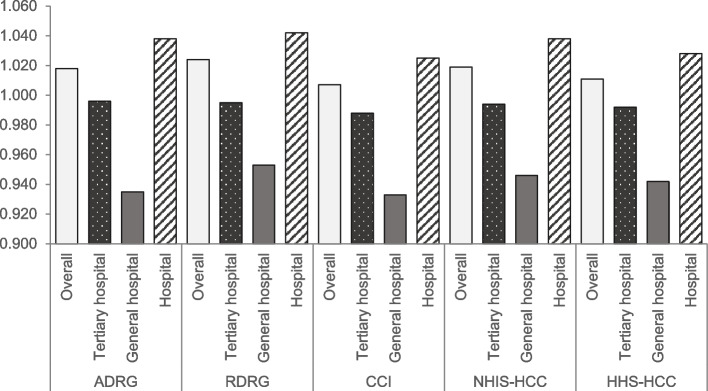
NSPE index according to institution type. ADRG, Adjacent Diagnosis Related Group; CCI, Charlson Comorbidity Index; HHS-HCC, Department of Health and Human Service Hierarchical Condition Category; NHIS-HCC, National Health Insurance Service Hierarchical Condition Category; NSPE, National Health Insurance Service Spending Per Episode; RDRG, Refined Diagnosis Related Group

Utilizing predicted values from individual models, we calculated the NSPE indexes and presented according to the institution type (Table 5, Fig. 7). The average NSPE indexes were above 1 in all models, suggesting that the average efficiency is worse than the benchmark institution representing the median value. Among the three types of institution, the efficiency values were superior in general hospitals and inferior in hospitals in all models. The average NSPE index was the highest (1.024) in Model 1 using RDRG and the lowest (1.007) in Model 2 using CCI (Table 5). Regarding the distribution of NSPE indexes, Model 2 showed the most narrow distribution (SD, 0.350), whereas Model 0 had the widest distribution (SD, 0.370). The range of NSPE indexes was higher in Model 3 (5.177) than in other models.

**Table 5:** Comparison of NSPE index between models

Model	Variables	N	Mean (SD)	Min	Q1	Median	Q3	Max	IQR	Range
Model 0 (ADRG)	Overall	1,665	1,018 (0.370)	0.005	0.825	0.978	1.136	4.550	0.311	4.545
	Tertiary hospital	42	0.996 (0.072)	0.781	0.951	0.999	1.043	1.181	0.091	0.400
	General hospital	310	0.935 (0.208	0.425	0.803	0.916	1.043	2.422	0.241	1.997
	Hospital	1,313	1.038 (0.401)	0.005	0.826	0.997	1.187	4.550	0.361	4.545
Model 1 (RDRG)	Overall	1,665	1.024 (0.363)	0.005	0.843	0.990	1.135	4.629	0.292	4.624
	Tertiary hospital	42	0.995 (0.075)	0.855	0.934	0.987	1.050	1.174	0.116	0.318
	General hospital	310	0.953 (0.203)	0.406	0.837	0.945	1.041	2.222	0.205	1.817
	Hospital	1,313	1.042 (0.395)	0.005	0.841	1.008	1.174	4.629	0.332	4.624
Model 2 (ADRG + CCI)	Overall	1,665	1.007 (0.350)	0.005	0.821	0.976	1.127	3.619	0.306	3.613
	Tertiary hospital	42	0.988 (0.071)	0.810	0.940	0.981	1.037	1.148	0.097	0.338
	General hospital	310	0.933 (0.202)	0.420	0.801	0.927	1.031	2.222	0.230	1.802
	Hospital	1,313	1.025 (0.379)	0.005	0.822	0.994	1.162	3.619	0.340	3.613
Model 3 (ADRG + NHIS-HCC)	Overall	1,665	1.019 (0.368)	0.024	0.835	0.987	1.130	5.201	0.295	5.177
	Tertiary hospital	42	0.994 (0.068)	0.843	0.947	0.996	1.032	1.180	0.084	0.337
	General hospital	310	0.946 (0.197)	0.449	0.824	0.936	1.040	2.266	0.216	1.817
	Hospital	1,313	1.038 (0.401)	0.024	0.829	1.001	1.170	5.201	0.341	5.177
Model 4 (ADRG + HHS-HCC)	Overall	1,665	1.011 (0.354)	0.006	0.826	0.979	1.132	4.182	0.306	4.176
	Tertiary hospital	42	0.992 (0.070)	0.846	0.949	0.992	1.040	1.156	0.091	0.310
	General hospital	310	0.942 (0.197)	0.409	0.819	0.944	1.039	2.276	0.220	1.867
	Hospital	1,313	1.028 (0.385)	0.006	0.819	0.996	1.163	4.182	0.344	4.176

*ADRG* Adjacent Diagnosis Related Group, *CCI* Charlson Comorbidity Index, *HHS-HCC* Department of Health and Human Service Hierarchical Condition Category, *IQR* Interquartile Range, *NHIS-HCC* National Health Insurance Service Hierarchical Condition Categories, *NSPE* National Health Insurance Service Spending Per Episode, *RDRG* Refined Diagnosis Related Group, *SD* Standard Deviation, *Q1* Quartile 1, *Q3* Quartile 3

## Discussion

Our study provided meaningful evidence on the risk adjustment of episode-based costs reflecting recent interest in cost containment and efficiency measurement. First, our results support a fundamental principle in risk adjustment: the choice of risk adjustment methods should be made based on the outcome of interest [11]. The model using CCI (developed for mortality adjustment) did not show any superiority to risk adjustment methods specific to cost estimation, though it showed subtle improvement compared to the model not adjusted for comorbidities (Not adjusted adj. R^2^ 40.8%, CCI adj. R^2^ 42.7%, methods specific to cost estimation adj. R^2^ 45.8%–46.3%; Table 3). Second, HCCs were preferable methods in efficiency measurement to RDRG. Overall explanatory powers were higher in the HCC models (CCI adj. R^2^ 42.7%, RDRG adj. R^2^ 45.8%, NHIS-HCC adj. R^2^ 46.3%, HHS-HCC adj. R^2^ 45.9%; Table 3). Although the value of MAE was the smallest in the RDRG model (CCI MAE $1,158, RDRG MAE $1,099, NHIS-HCC MAE $1,126, HHS-HCC MAE $1,129; Fig. 3), RDRG does not differentiate complications and comorbidities for risk adjustment in the current KDRG system [23]. In addition, good model fits of RDRG are more likely due to the application of RDRG in seven diseases to determine payment within the KDRG-based payment system [40]. Third, we introduced HHS-HCC in the context of South Korea due to the limitation of NHIS-HCC targeting the older population [18, 33]. Adjustment methods should be comprehensive, given the purpose of risk adjustment for hospital efficiency measurement. Although NHIS-HCC showed its validity in several studies in South Korea [15–17], it does not precisely fit into the quality evaluation of hospitals due to the limited coverage of diseases. Hospitals providing a large volume of obstetric or pediatric services can have disadvantages in the evaluation. Fourth, our research design focuses on a pragmatic approach. Although various studies showed the superiority of HCCs, they evaluate the model performance based on annual costs. Depending on the reimbursement system, cost estimation can be annual, episode unit, etc. The factors contributing to cost rise can differ depending on the cost unit. Therefore, our strength is that our models are based on episode unit costs considering their actual utilization.

According to MDC groups, we observed similar performance patterns in each model to previous research using DRGs (Centers for Medicare and Medicaid Services Diagnosis Related Groups, CMS-DRG; Consolidated Severity-Adjusted DRGs, Con-APR DRG; Medicare Severity Diagnosis Related Groups, MS-DRG; RDRG). As in prior studies [41, 42], all models showed higher explanatory powers in MDC F (Diseases and Disorders of the Circulatory System, adj. R^2^ 60.2%–63.3%) and MDC I (Diseases and Disorders of the Musculoskeletal System and Connective Tissue, adj. R^2^ 54.1%–61.1%) than in the other MDC groups (Fig. 2). MDC UV (Mental Diseases and Disorders, adj. R^2^ 7.7%–12.1%) also followed previous research outcomes with the lowest explanatory power. In terms of MDC P, even the unadjusted model (adj. R^2^ 77.1%), including only ADRGs, described a relatively better performance of over 70%. However, the RDRG model (adj. R^2^ 77.1%) did not show improvement in model fits compared to the unadjusted model. The same number of code types between ADRG (*n* = 26) and RDRG (*n* = 26) implies that the KDRG system does not risk adjusting in MDC P.

There are several limitations in our study. First, we could not obtain enough time period to define the index admission and the lookback period to identify comorbidities due to the cross-sectional dataset of the HIRA-NPS [21]. Due to the confined index admission (between April and November), seasonal variation in the epidemiological data cannot be considered [43]. The longitudinal dataset might be a fundamental solution to issues defining the time period. Additionally, Present on admission (POA) indicators can be a strategy for using claims data efficiently. Although the current Korean health insurance system does not provide POA indicators for research, they differentiate comorbidities and complications in the claims data [44]. Therefore, the use of POA indicators can reduce the lookback period. Second, we used HCCs based on the Korean modification 7th of the ICD-10 (KCD-7), which were transformed from the versions developed in the International Classification of Diseases, Ninth Revision, Clinical Modification (ICD-9-CM) and the International Classification of Diseases, Tenth Revision, Clinical Modification (ICD-10-CM). Therefore, information loss is inevitable during the transformation process due to the limited transferability of ICD codes between countries. In particular, the ICD-9-CM or ICD-10-CM coding systems are more fragmented due to the inclusion of procedure codes [45]. Third, the Korean claims system only collects the payer’s amount and a portion of the out-of-pocket cost (i.e., statutory payment by the patient) but does not include non-payment items by the payer. According to the benefit coverage rate survey, non-payment items comprised 15.6% of the total annual expenditure 2018 [46]. In addition, the proportion of non-payment items varied depending on institutional types and disease groups. For example, while non-payment items of hospitals accounted for 33.0%, tertiary and general hospitals accounted for 11.4% and 11.6%, respectively [46]. Furthermore, depending on disease groups, non-payment items ranged from 0.4% in human immunodeficiency virus disease to 22.9% in malignant neoplasms of female genital organs [46]. These differences suggest that total cost might differ after including non-payment items between MDC groups.

There are still opportunities to improve models by introducing sophisticated statistical methods in further studies. Our study tried to tackle the skewed distribution in the sensitivity analyses. After observing improved distribution by winsorizing the cost at 0.5 percentile (Additional file 5), the winsorized costs were used in our basic models. We also explored the log-transformation and trimming techniques. Regarding log transformation, performance improvement was observed in all MDC groups except MDC P (Fig. 4). On the other hand, a reduction in explanatory power in several MDCs (MDC P, MDC R, MDC ST, MDC UV, and MDC WXY) might have implied significant information loss in trimming at IQR (Fig. 4). We confirmed tentative conclusions, such as the benefits of using winsorized cost and the inappropriateness of trimming. Nevertheless, more rigorous statistical techniques should be covered to deal with skewed cost data in further studies, such as weighted least squares, the Generalized Linear Model (GLM) with gamma distribution, and constrained regression [14, 47, 48]. Additionally, we explored the clustering effects regarding types of medical institutions. The ICCs (0.018–0.500) suggest that costs from different institutional types were more discrepant from one another than the costs within the types of hospitals (Additional file 6). Our multilevel analysis results suggest further investigation into clustering effects. Inferior model performance in MDC I (the largest AIC and BIC) differs from our basic model using linear regression and the previous research comparing performance between MDC groups. The basic OLS regression models included institution types as independent variables considering the Korean Reource-Based Relative Value Scale (RBRVS) weighting scheme. Within the Korean RBRVS scheme, services in upper-level hospitals are reimbursed higher than in lower-level institutions [49]. There might be little difference between types of hospitals in a single insurer system like South Korea, except for service types and comorbidities. More studies need to investigate clustering effects on cost estimation within the context of the insurance system.

## Conclusions

Our results suggest using risk adjustment methods specific to costs, such as HCCs, rather than CCI or risk-adjusted DRG in episode-based efficiency measurements. However, the subtle difference between the two HCCs suggests that more studies are needed to evaluate and further tailor them. Nevertheless, with recent increasing attention to efficiency, our methods and results can contribute to adopting and scaling up efficiency measures in the value-based payment system.

### Supplementary Information

**Additional file 1.** Adjustment rules for overlapped episode windows.

**Additional file 2.** Comparison of MDCs between the original version of KDRG and the modified version for this study.

**Additional file 3.** Winsorizing and trimming cutoffs according to the MDC.

**Additional file 4.** Regression coefficients and multicollinearity test results.

**Additional file 5.** Histograms of residuals of NSPE episode costs according to the MDC.

**Additional file 6.** The results of multilevel analysis.

## Data Availability

All available data can be obtained by contacting the corresponding author.

## Abbreviations

ADRG: Adjacent diagnosis related group
AIC: Akaike information criterion
BIC: Schwarz’s Bayesian information criterion
CCI: Charlson comorbidity index
CMS: Center for Medicare and Medicaid Services
CMS-DRG: Centers for Medicare and Medicaid Services Diagnosis Related Groups
CMS-HCC: Center for Medicare and Medicaid Services hierarchical condition categories
Con-APR DRG: Consolidated severity-adjusted diagnosis-related group
DRG: Diagnosis-related group
ER: Emergency room
GDP: Gross domestic product
GLM: Generalized linear model
HCC: Hierarchical condition categories
HHS-HCC: Department of health and human service hierarchical condition categories
HIRA: Health Insurance Review and Assessment Service
HIRA-NPS: Health Insurance Review and Assessment Service-national patient sample
ICC: Instracluster correlation coefficient
ICD-10-CM: International classification of diseases, tenth revision, clinical modification
ICD-9-CM: International classification of diseases, ninth revision, clinical modification
IQR: Interquartile range
KCD-7: Korean modification 7th of the international classification of diseases, tenth revision
KRW: South Korean won
MAE: Mean absolute error
MDC: Major diagnostic category
MS-DRG: Medicare severity diagnosis related groups
NHIS: National Health Insurance Service
NHIS-HCC: National health insurance service hierarchical condition categories
NSPE: National Health Insurance Service spending per episode
OECD: Organisation for Economic Cooperation and Development
OLS: Ordinary least squares
POA: Present on admission
PR: Predictive ratio
R^2^: R-squared
RDRG: Refined diagnosis related group
RBRVS: Reource-based relative value scale
SD: Standard deviation
USD: United States dollars
