# Supplementary material for: Comparison of diagnosis-based risk adjustment methods for episode-based costs to apply in efficiency measurement
Source: BMC Health Serv Res. 2023 Dec 1;23:1334. doi: 10.1186/s12913-023-10282-4 (PMC10693049; doi:10.1186/s12913-023-10282-4)
Supplement: Supplementary file 1 — Additional file 1. Adjustment rules for overlapped episode windows. [file 12913_2023_10282_MOESM1_ESM.pptx]

## Slide 1
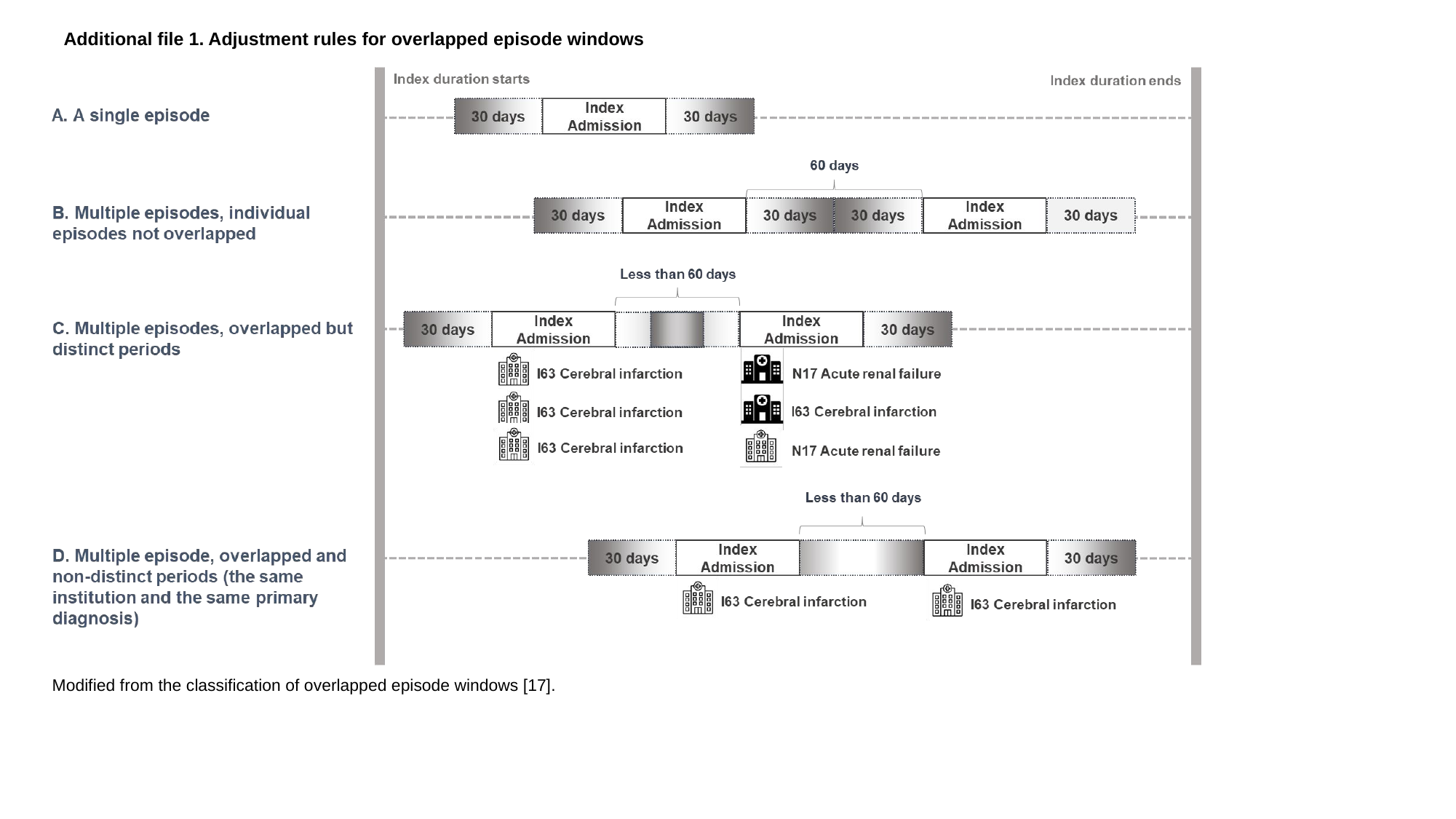

Additional file 1. Adjustment rules for overlapped episode windows
Modified from the classification of overlapped episode windows [17].
