# Supplementary material for: Comparison of diagnosis-based risk adjustment methods for episode-based costs to apply in efficiency measurement
Source: BMC Health Serv Res. 2023 Dec 1;23:1334. doi: 10.1186/s12913-023-10282-4 (PMC10693049; doi:10.1186/s12913-023-10282-4)
Supplement: Supplementary file 2 — Additional file 2. Comparison of MDCs between the original version of KDRG and the modified version for this study. [file 12913_2023_10282_MOESM2_ESM.docx]

# Additonal file 2. Comparison of MDCs between the original version of KRDG and the modified version for this study

| Original version^a^ | MDC name | Modified version | MDC name |
| --- | --- | --- | --- |
| MDC A | PreMDC | Excluded |  |
| MDC B | Diseases and Disorders of the Nervous System | MDC B | Diseases and Disorders of the Nervous System |
| MDC C | Diseases and Disorders of the Eye | MDC C | Diseases and Disorders of the Eye |
| MDC D | Diseases and Disorders of the Ear, Nose, Mouth and Throat | MDC D | Diseases and Disorders of the Ear, Nose, Mouth and Throat |
| MDC E | Diseases and Disorders of the Respiratory System | MDC E | Diseases and Disorders of the Respiratory System |
| MDC F | Diseases and Disorders of the Circulatory System | MDC F | Diseases and Disorders of the Circulatory System |
| MDC G | Diseases and Disorders of the Digestive System | MDC G | Diseases and Disorders of the Digestive System |
| MDC H | Diseases and Disorders of the Hepatobiliary System and Pancreas | MDC H | Diseases and Disorders of the Hepatobiliary System and Pancreas |
| MDC I | Diseases and Disorders of the Musculoskeletal System and Connective Tissue | MDC I | Diseases and Disorders of the Musculoskeletal System and Connective Tissue |
| MDC J | Diseases and Disorders of the Skin, Subcutaneous Tissue and Breast | MDC J | Diseases and Disorders of the Skin, Subcutaneous Tissue and Breast |
| MDC K | Endocrine, Nutritional and Metabolic Diseases and Disorders | MDC K | Endocrine, Nutritional and Metabolic Diseases and Disorders |
| MDC L | Diseases and Disorders of the Kidney and Urinary Tract | MDC L | Diseases and Disorders of the Kidney and Urinary Tract |
| MDC M | Diseases and Disorders of the Male Reproductive System | MDC M | Diseases and Disorders of the Male Reproductive System |
| MDC N | Diseases and Disorders of the Female Reproductive System | MDC N | Diseases and Disorders of the Female Reproductive System |
| MDC O | Pregnancy, Childbirth and Puerperium | MDC O | Pregnancy, Childbirth and Puerperium |
| MDC P | Newborns and Other Neonates | MDC P | Newborns and Other Neonates |
| MDC Q | Diseases and Disorders of the Blood and Blood-Forming Organs and Immunological Disorders | Excluded | Diseases and Disorders of the Blood and Blood-Forming Organs and Immunological Disorders |
| MDC R | Neoplastic Disorders (Haematological and Solid Neoplasms) | MDC R | Neoplastic Disorders (Haematological and Solid Neoplasms) |
| MDC S | Infectious and Parasitic Diseases: HIV | MDC ST | Infectious and Parasitic Diseases |
| MDC T | Infectious and Parasitic Diseases |  |  |
| MDC U | Mental Diseases and Disorders | MDC UV | Mental Diseases and Disorders |
| MDC V | Alcohol/Drug Use and Alcohol/Drug Induced Organic Mental Disorders |  |  |
| MDC W | Multiple Trauma | MDC WXY | Trauma, Injuries, Poisoning and Burns |
| MDC X | Injuries, Poisoning and Toxic Effects of Drugs |  |  |
| MDC Y | Burns |  |  |
| MDC Z | Factors Influencing Health Status and Other Contacts with Health Services | Excluded |  |

^a^KDRG Ver 4.2 [23].
KDRG, Korean Diagnostic Related Group; MDC, Major Diagnostic Category.
