# Supplementary material for: Comparison of diagnosis-based risk adjustment methods for episode-based costs to apply in efficiency measurement
Source: BMC Health Serv Res. 2023 Dec 1;23:1334. doi: 10.1186/s12913-023-10282-4 (PMC10693049; doi:10.1186/s12913-023-10282-4)
Supplement: Supplementary file 3 — Additional file 3. Winsorizing and trimming cutoffs according to the MDC. [file 12913_2023_10282_MOESM3_ESM.docx]

**Additional file 3. Winsorizing and trimming cutoffs according to the MDC**

| **MDC** | **2017^a^** | | | | **2018^b^** | | | |
| --- | --- | --- | --- | --- | --- | --- | --- | --- |
|  | **0.5 pct** | **Q1** | **Q75** | **99.5 pct** | **0.5 pct** | **Q1** | **Q75** | **99.5 pct** |
| B | 110 | 644 | 2,809 | 30,284 | 123 | 743 | 3,245 | 34,747 |
| C | 104 | 1,023 | 2,339 | 6,941 | 126 | 1,174 | 2,614 | 6,840 |
| D | 88 | 394 | 1,119 | 9,940 | 93 | 442 | 1,293 | 10,246 |
| E | 151 | 585 | 2,157 | 20,410 | 175 | 627 | 2,332 | 25,414 |
| F | 80 | 967 | 5,730 | 38,469 | 146 | 1,084 | 6,306 | 42,581 |
| G | 111 | 609 | 1,998 | 15,463 | 117 | 691 | 2,203 | 17,433 |
| H | 149 | 1,193 | 3,745 | 22,144 | 202 | 1,319 | 4,279 | 28,454 |
| I | 84 | 502 | 2,252 | 12,515 | 80 | 534 | 2,521 | 14,411 |
| J | 65 | 569 | 2,090 | 13,048 | 65 | 613 | 2,413 | 16,770 |
| K | 32 | 678 | 2,299 | 12,175 | 30 | 851 | 2,641 | 15,772 |
| L | 161 | 925 | 2,748 | 22,614 | 184 | 1,069 | 3,120 | 23,582 |
| M | 119 | 812 | 2,311 | 15,784 | 128 | 877 | 2,697 | 15,609 |
| N | 104 | 918 | 2,870 | 12,590 | 128 | 1,017 | 3,345 | 14,021 |
| O | 148 | 1,004 | 1,690 | 5,975 | 136 | 1,154 | 1,891 | 7,788 |
| P | 57 | 165 | 729 | 42,677 | 73 | 230 | 1,074 | 51,345 |
| R | 159 | 954 | 3,273 | 45,360 | 126 | 1,069 | 3,427 | 49,567 |
| ST | 88 | 412 | 1,541 | 23,358 | 80 | 462 | 1,687 | 27,040 |
| UV | 81 | 571 | 3,095 | 15,186 | 103 | 632 | 3,364 | 17,558 |
| WXY | 125 | 723 | 2,509 | 26,744 | 125 | 792 | 2,765 | 24,544 |

^a^Unit: United States Dollar (USD), converted from South Korean Won (KRW) (1 USD = 1,130.48 KRW, 2017); ^b^Unit: United States Dollar (USD), converted from South Korean Won (KRW) (1 USD = 1,100.58 KRW, 2018).
MDC, Major Diagnostic Category; pct, Percentile. Q1, Quartile 1; Q3, Quartile 3.
