# Supplementary material for: Comparison of diagnosis-based risk adjustment methods for episode-based costs to apply in efficiency measurement
Source: BMC Health Serv Res. 2023 Dec 1;23:1334. doi: 10.1186/s12913-023-10282-4 (PMC10693049; doi:10.1186/s12913-023-10282-4)
Supplement: Supplementary file 5 — Additional file 5. Histograms of residuals of NSPE episode costs according to the MDC. [file 12913_2023_10282_MOESM5_ESM.docx]

**Additional file 5. Histograms of residuals of NSPE episode costs according to the MDC**

| **MDC** | **Original cost** | **Winsorized cost at 0.5 percentile** | **Log-transformed cost** | **Trimmed cost using IQR method** |
| --- | --- | --- | --- | --- |
| B | 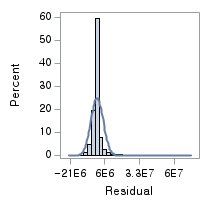 | 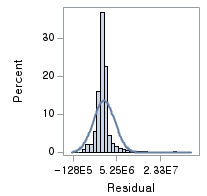 | 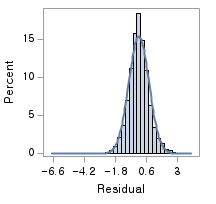 | 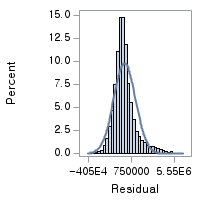 |
| C | 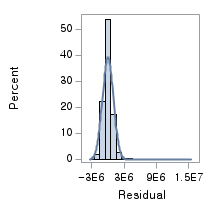 | 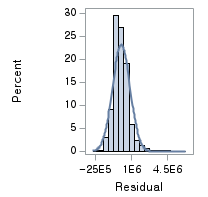 | 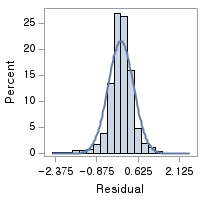 | 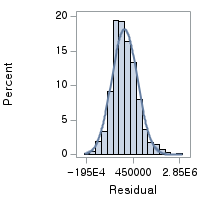 |
| D | 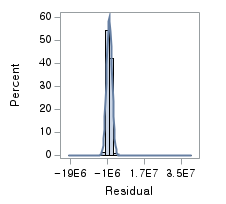 | 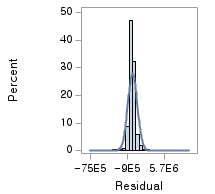 | 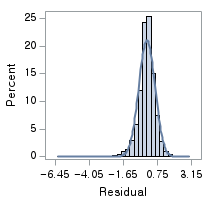 | 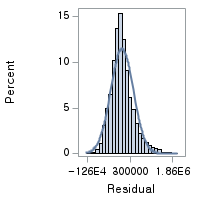 |
| E | 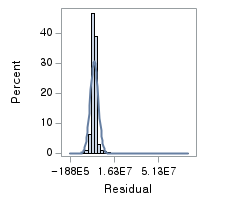 | 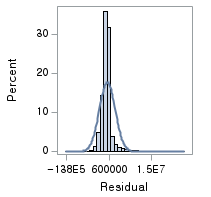 | 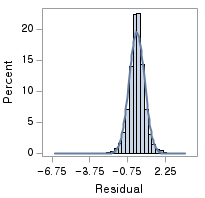 | 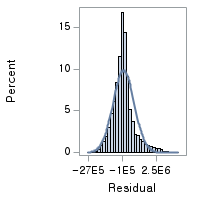 |
| F | 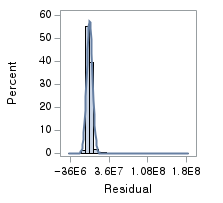 | 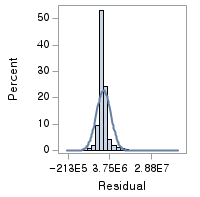 | 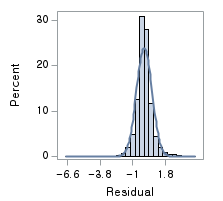 | 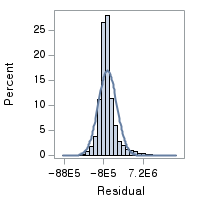 |
| G | 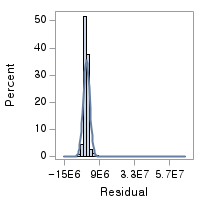 | 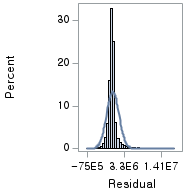 | 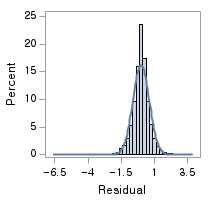 | 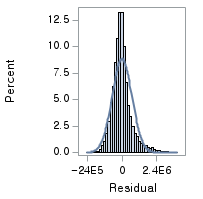 |
| H | 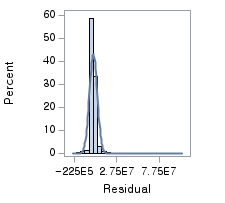 | 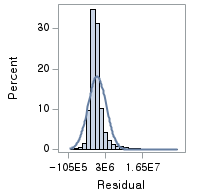 | 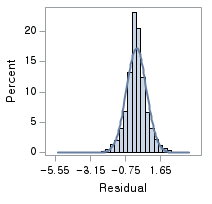 | 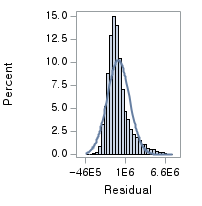 |
| I | 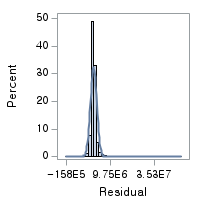 | 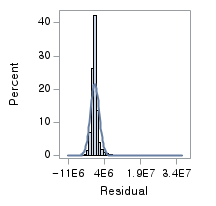 | 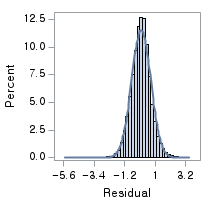 | 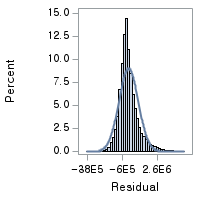 |
| J | 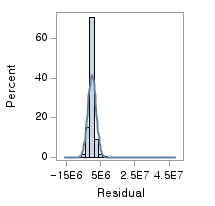 | 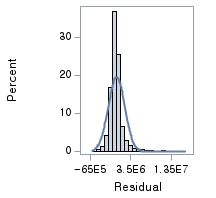 | 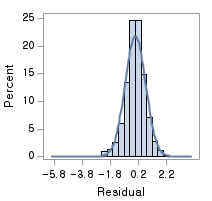 | 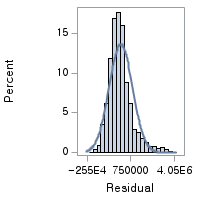 |
| K | 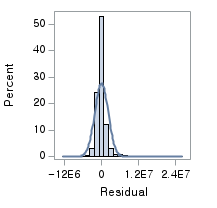 | 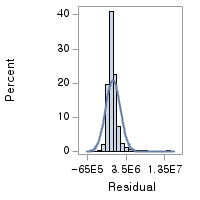 | 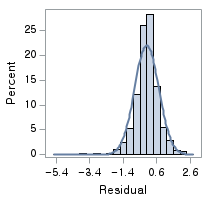 | 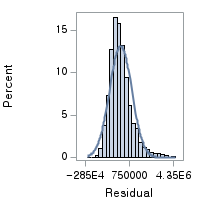 |
| L | 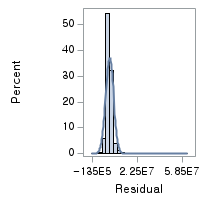 | 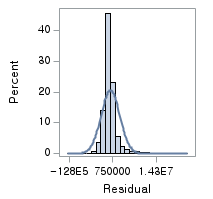 | 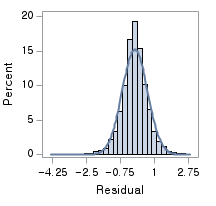 | 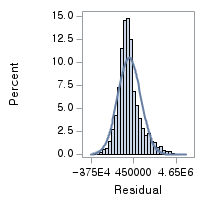 |
| M | 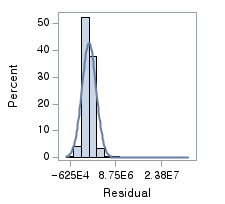 | 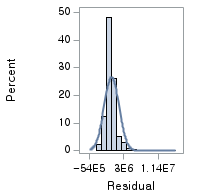 | 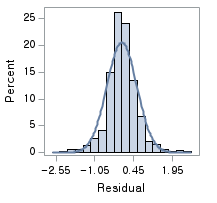 | 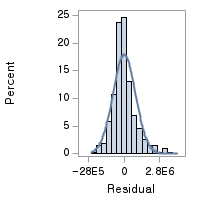 |
| N | 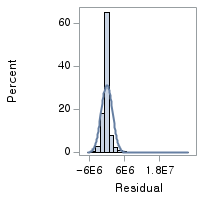 | 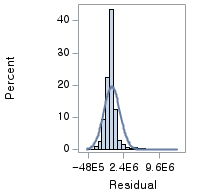 | 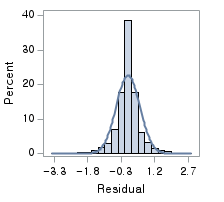 | 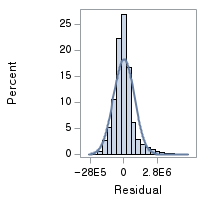 |
| O | 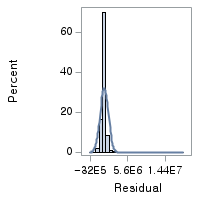 | 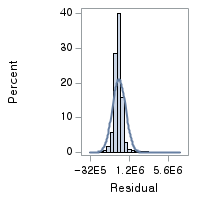 | 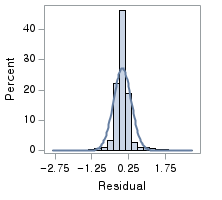 | 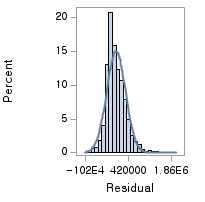 |
| P | 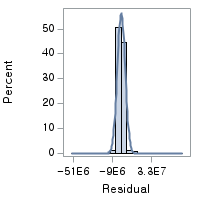 | 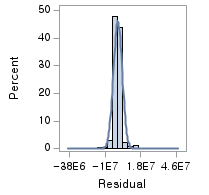 | 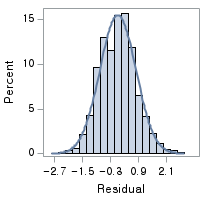 | 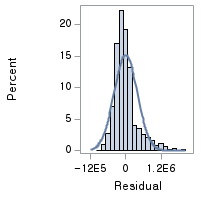 |
| R | 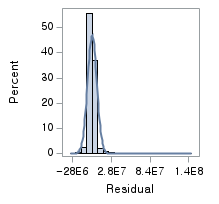 | 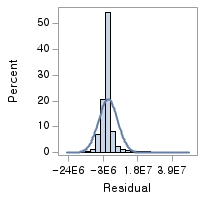 | 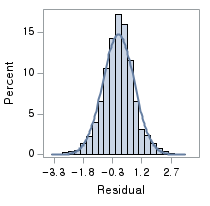 | 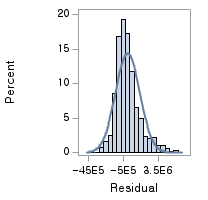 |
| ST | 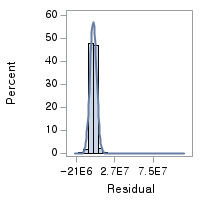 | 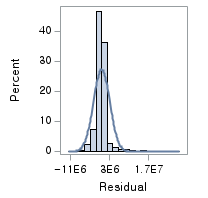 | 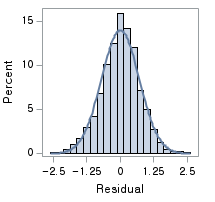 | 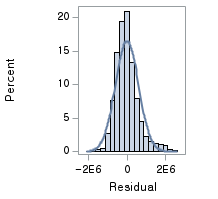 |
| UV | 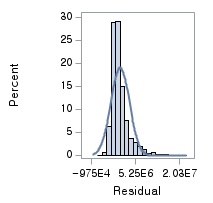 | 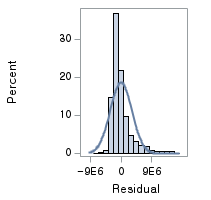 | 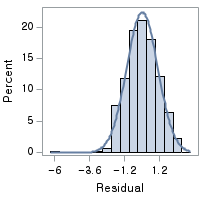 | 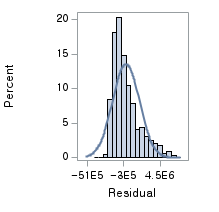 |
| WXY | 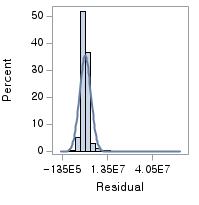 | 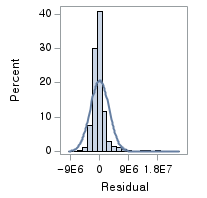 | 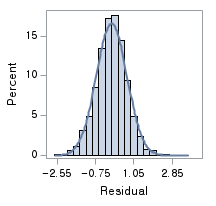 | 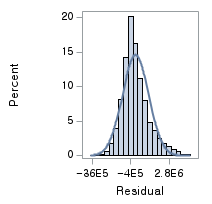 |

IQR, Interquartile Range; MDC, Major Diagnostic Category; NSPE, National Health Insurance Service Spending Per Episode.
